# Mutations in *CDCA7* and *HELLS* cause immunodeficiency–centromeric instability–facial anomalies syndrome

**DOI:** 10.1038/ncomms8870

**Published:** 2015-07-28

**Authors:** Peter E. Thijssen, Yuya Ito, Giacomo Grillo, Jun Wang, Guillaume Velasco, Hirohisa Nitta, Motoko Unoki, Minako Yoshihara, Mikita Suyama, Yu Sun, Richard J. L. F. Lemmers, Jessica C. de Greef, Andrew Gennery, Paolo Picco, Barbara Kloeckener-Gruissem, Tayfun Güngör, Ismail Reisli, Capucine Picard, Kamila Kebaili, Bertrand Roquelaure, Tsuyako Iwai, Ikuko Kondo, Takeo Kubota, Monique M. van Ostaijen-Ten Dam, Maarten J. D. van Tol, Corry Weemaes, Claire Francastel, Silvère M. van der Maarel, Hiroyuki Sasaki

**Affiliations:** 1 Department of Human Genetics, Leiden University Medical Center, Leiden 2333ZA, The Netherlands; 2 Division of Epigenomics and Development, Department of Molecular Genetics, Medical Institute of Bioregulation, Kyushu University, Fukuoka 812-8582, Japan; 3 CNRS UMR7216, Epigenetics and Cell Fate, Université Paris Diderot, Sorbonne Paris Cité, 75205 Paris, France; 4 Division of Bioinformatics, Department of Multi-scale Research Center for Medical Science, Medical Institute of Bioregulation, Kyushu University, Fukuoka 812-8582, Japan; 5 Department of Paediatric Immunology, Newcastle Upon Tyne Hospital, NHS Foundation Trust, Newcastle Upon Tyne, UK; 6 Institute of Cellular Medicine, Newcastle University, Newcastle Upon Tyne NE1 4LP, UK; 7 Division of Pediatrics and Pediatric Rheumatology, G. Gaslini Scientific Institute, Genova 16147, Italy; 8 Institute of Medical Molecular Genetics, University of Zurich, Schlieren 8952, Switzerland; 9 Department of Biology, ETH Zurich, Zurich 8093, Switzerland; 10 Department of Oncology, University Children’s Hospital, Zurich 8032, Switzerland; 11 Department of Pediatric Immunology and Allergy, Necmettin Erbakan University, Meram Medical Faculty, Konya 42080, Turkey; 12 Centre de Référence Déficits Immunitaires Héréditaires, AP-HP, 75743 Paris, France; 13 Centre d’Etude des Déficits Immunitaires, Hôpital Universitaire Necker-Enfants Malades, AP-HP, 75743 Paris, France; 14 Laboratoire de Génétique Humaine des Maladies Infectieuses, Inserm, 75743, Paris, France; 15 Université Paris Descartes, Institut Imagine, Sorbonne Paris, 75743 Paris, France; 16 Unité d'immunologie et d’hématologie pédiatrique, Hôpital Necker-Enfants Malades, 75743, Paris, France; 17 Inserm UMR 768, 75015 Paris, France; 18 Centre de Référence Déficits Immunitaires Héréditaires, Institut d’Hématologie et d’Oncologie Pédiatrique, CHU de Lyon, 69008 Lyon, France; 19 Service d’hépato-gastro-entérologie et nutrition, endocrinologie et néphrologie pédiatriques, Hôpital de la Timone, CHU Marseille, 13385 Marseille, France; 20 Department of Pediatric Hematology and Oncology, Shikoku Medical Center for Children and adults, Kagawa 765-8507, Japan; 21 Department of Pediatrics, Ooida Hospital, Kochi 788-0001, Japan; 22 Department of Epigenetic Medicine, Faculty of Medicine, Interdisciplinary Graduate School of Medicine and Engineering, University of Yamanashi, Yamanashi 409-3898, Japan; 23 Department of Pediatrics, Laboratory for Immunology, Leiden University Medical Center, Leiden 2333ZA, The Netherlands; 24 Department of Pediatric Infectious Diseases and Immunology, Radboud University Nijmegen Medical Center, Nijmegen 6500HC, The Netherlands

## Abstract

The life-threatening Immunodeficiency, Centromeric Instability and Facial Anomalies (ICF) syndrome is a genetically heterogeneous autosomal recessive disorder. Twenty percent of patients cannot be explained by mutations in the known ICF genes DNA methyltransferase 3B or zinc-finger and BTB domain containing 24. Here we report mutations in the cell division cycle associated 7 and the helicase, lymphoid-specific genes in 10 unexplained ICF cases. Our data highlight the genetic heterogeneity of ICF syndrome; however, they provide evidence that all genes act in common or converging pathways leading to the ICF phenotype.

## Abstract (web summary)

Immunodeficiency-centromeric instability-facial anomalies syndrome is a life threatening autosomal recessive disorder caused by mutations in *DNMT3B* and *ZBTB24*. Here Thijssen *et al.* identify mutations in *CDCA7* and *HELLS* in previously unexplained cases.

## Graphical abstract

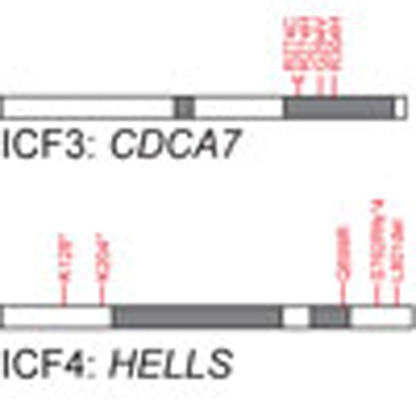

Immunodeficiency, Centromeric Instability and Facial Anomalies (ICF) syndrome is characterized by recurrent and often fatal respiratory and gastrointestinal infections as a consequence of hypo- or a-gammaglobulinemia in the presence of B cells^1^,^2^. Nearly all patients also present with distinct facial anomalies, including hypertelorism, flat nasal bridge and epicanthus^1^,^2^. Centromeric instability is the cytogenetic hallmark of ICF syndrome. It involves the juxtacentromeric heterochromatin repeats on chromosomes 1, 9 and 16 and is comparable to what is observed after treatment of cells with demethylating agents^3^,^4^. Therefore, CpG hypomethylation of juxtacentromeric satellite types II and III is a diagnostic for ICF syndrome, with additional hypomethylation of centromeric α-satellite repeats in *DNMT3B* mutation-negative patients^5^,^6^.

Mutations in the DNA methyltransferase 3B (*DNMT3B*) gene (OMIM 602900; ICF1) account for ∼50% of ICF cases, while ∼30% of cases have mutations in the zinc-finger and BTB domain containing 24 (*ZBTB24*) gene (OMIM 614064; ICF2)^2^,^5^. *DNMT3B* is a *de novo* DNA methyltransferase, primarily acting during early development with a preference for CpG dense regions^7^. ICF1 mutations in the catalytic domain of *DNMT3B* result in largely reduced methyltransferase activity^8^,^9^. The function of *ZBTB24* is unknown, but it belongs to a family of ZBTB proteins of which many have regulatory roles in haematopoietic differentiation^10^,^11^. Despite the successful identification of ICF genes, the pathophysiological mechanism underlying the syndrome remains largely unresolved.

Previous studies indicated further genetic heterogeneity in ICF syndrome^2^. To identify the genetic cause in genetically unexplained cases (ICFX), we combined homozygosity mapping with whole-exome sequencing. By using an autosomal recessive inheritance model and prioritizing homozygous variants in consanguineous families, we now identify four different homozygous and potentially damaging variants in the cell division cycle associated 7 (*CDCA7*) gene in five ICFX patients (now referred to as ICF3). In an additional five ICFX patients, we identify compound heterozygous and homozygous variants in the helicase, lymphoid-specific (*HELLS*) gene (ICF4). We show that knockdown of both new ICF genes leads to hypomethylation of juxtacentromeric heterochromatin repeats in a murine cell model. Our results emphasize the genetic heterogeneity of ICF syndrome, nonetheless providing evidence that all four ICF genes are involved in at least one common pathway.

## Results

### Missense mutations in *CDCA7* cause ICF syndrome type 3

**Figure 1:**
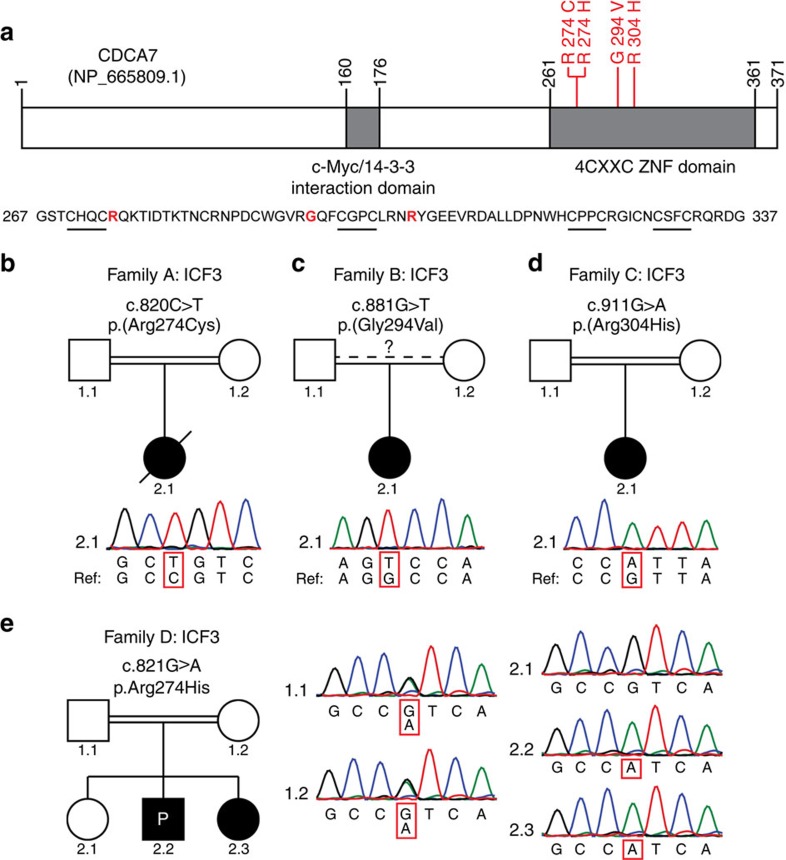
Homozygous missense mutations in *CDCA7* in five ICF3 patients. (**a**) Schematic representation of CDCA7, with the identified homozygous missense mutations in red. Sequence outtake: 4-CXXC-type zinc-finger domain, CXXC motifs are underlined, mutated residues in red. (**b**–**d**) Sanger sequencing confirmation of missense mutations in CDCA7 in families A–C. All variants were homozygous, the reference sequence is displayed for comparison. (**e**) Sanger sequencing confirmation of the homozygous missense CDCA7 mutation in patients 2.2 (proband) and 2.3 of family D. Both parents are heterozygous for the variant, sibling 2.1 is unaffected and homozygous for the wild-type (wt) allele.

We selected 13 ICFX patients from 11 families, negative for mutations in *DNMT3B* or *ZBTB24*, of whom the clinicopathological characteristics are listed in Supplementary Table 1. Hypomethylation of pericentromeric satellite type II (Sat II), common to all ICF patients, and centromeric α-satellite DNA repeats, shown to be affected only in ICF2 and ICFX, was shown before for a subset of patients^2^,^5^,^6^,^12^. For an additional set of ICFX patients, we confirmed Sat II and α-satellite hypomethylation using Southern blot analysis (Supplementary Fig. 1). In five ICFX patients from four families we identified and confirmed homozygous missense mutations in *CDCA7,* all near the first two zinc-finger motifs in the conserved carboxyterminal 4-CXXC-type zinc-finger domain (Fig. 1a–e). Segregation with disease was confirmed in family D and all mutations were predicted to be pathogenic and have an allele frequency supporting pathogenicity (Fig. 1e, Supplementary Table 1). *CDCA7* is involved in neoplastic transformation, *MYC*-dependent apoptosis and haematopoietic stem cell emergence; however, its molecular function is unknown^13^,^14^. All four zinc-finger motifs are completely conserved in the highly homologous 4-CXXC zinc-finger domain of the transcriptional repressor *CDCA7-Like* (*CDCA7L*; Supplementary Fig. 2). The repressive activity of CDCA7L is dependent on its 4-CXXC domain, and by homology *CDCA7* mutations in ICF3 may disrupt a similar function^15^.

### Mutations in HELLS cause ICF syndrome type 4

**Figure 2:**
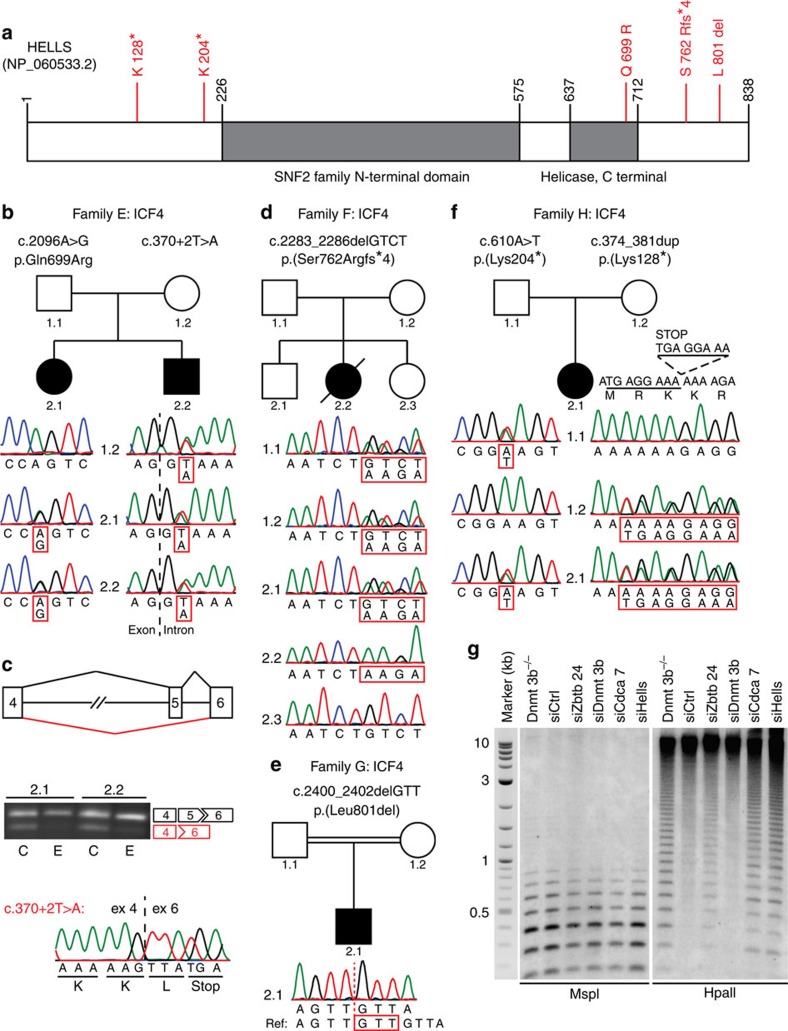
Mutations in *HELLS* in five ICF4 patients. (**a**) Schematic representation of HELLS, with the identified mutations in red. (**b**) Sanger sequencing confirmation of HELLS mutations in family E. Only c.370+2T>A was identified in maternal DNA, indicating different allelic origins of both mutations, or *de novo* occurrence of the second mutation. (**c**) RT–PCR analysis of HELLS mRNA on treatment of patient-derived fibroblasts with cycloheximide (C) revealed that c.370+2T>A leads to complete skipping of exon 5 and disruption of the open reading frame. Ethanol-treated samples (E) served as controls, alternative splicing was confirmed using Sanger’s sequencing in two independent experiments for both samples. (**d**) Sanger sequencing confirmation of a homozygous out-of-frame deletion in *HELLS* in family F. Both parents as well as unaffected sibling 2.1 are heterozygous for the deletion allele; unaffected sibling 2.3 is homozygous for the wt allele. (**e**) Sanger sequencing confirmation of a homozygous in-frame deletion in *HELLS* in family G. Both parents are heterozygous for the deletion allele. (**f**) Sanger sequencing confirmation of nonsense mutations in *HELLS* in family H. Different allelic origin was confirmed in parental DNA. (**g**) Southern blot analysis of minor satellite DNA methylation in *Dnmt3b*^−/−^ and siRNA-treated wt MEFs after digesting DNA with MspI or its methylation-sensitive isoschizomer HpaII revealed CpG hypomethylation on knockdown of Zbtb24, Cdca7 and Hells. Molecular weights of the 2-Log DNA size marker are in kilobasepairs.

In an additional five ICFX patients from four families, we identified mutations in *HELLS* that were predicted to be pathogenic and with allele frequencies supporting pathogenicity (Fig. 2a, Supplementary Table 1, ICF4). In affected members of family E, we identified a missense mutation in the conserved helicase domain (c.2096A>G;p.[Gln699Arg]) and an intronic mutation leading to destruction of the splice donor site in intron 5 (c.370+2T>A; Fig. 2b). Different allelic origin is supported by analysis of maternal DNA, which carried only the splice site mutation (Fig. 2c). To analyse the effect of the lost splice donor site on mRNA processing, fibroblasts of both patients were treated with cycloheximide to inhibit nonsense-mediated decay. Upon treatment, reverse transcriptase–PCR (RT–PCR) analysis showed increased levels of a splice variant with complete skipping of exon 5, leading to a frameshift followed by a premature stop codon in exon 6 (Fig. 2c).

The patient in family F carries a homozygous out-of-frame deletion (c.2283_2286delGTCT;p.[Ser762Argfs*4]), resulting in a frameshift and introduction of a premature stop codon in exon 20. The unaffected siblings 2.1 and 2.3 were found to be heterozygous for the deletion or homozygous for the wild-type allele, respectively (Fig. 2d). In family G we found a deleterious homozygous in-frame deletion in the C-terminal domain of *HELLS*, leading to the deletion of Leucine 801 (c.2400_2402delGTT;p.[Leu801del]; Fig. 2e). The proband of family H carries a nonsense mutation (c.610A>T;p.[Lys204*]) and a duplication causing the insertion of a stop codon (c.374_381dup;p.[Lys128*]), suggesting that absence of HELLS is compatible with human life, whereas *Hells*^−/−^ mice die perinatally^16^. Different allelic origin of the mutations was confirmed in parental DNA (Fig. 2f).

### ICF genes converge at centromeric DNA methylation regulation

In mouse, HELLS is required for T-cell proliferation and mediates *de novo* DNA methylation, through its interaction with DNMT3B, dependent on its ATPase domain^16^,^17^,^18^,^19^. Genome-wide loss of CpG methylation, including centromeric repeats, has been observed in *Hells*^−/−^ mice, reminiscent of what has been described in *Dnmt3b* knockout mice and in mouse models for ICF1 (refs 20, 21). We show that transient depletion of HELLS, CDCA7 and ZBTB24, but not DNMT3B, resulted in decreased CpG methylation at centromeric repeats in wild-type mouse embryonic fibroblasts (MEFs, Fig. 2g, Supplementary Fig 3a,b). This confirms that DNMT3B acts during establishment of centromeric CpG methylation^22^. Moreover, it supports a role for ZBTB24 and CDCA7 in maintenance of CpG methylation at centromeric repeats, and, combined with previously published work, suggests that HELLS may be involved in both processes^19^.

By identifying two new ICF syndrome genes this study highlights the genetic heterogeneity of the syndrome. The identification of at least one additional disease gene is expected with still a few cases remaining genetically unresolved. The complex, but highly overlapping, pathophysiology suggests that all ICF genes act in common or converging pathways involved in immunity, chromatin regulation and development. Convergence is supported by hypomethylation of pericentromeric repeats, which is common to all ICF subgroups, however the result of different defective pathways in the establishment and/or maintenance of CpG methylation.

## Methods

### Patients

All samples were obtained in an anonymized manner, and all families gave consent for genetic analyses. The study was approved by the Medical Ethical Committee of the Leiden University Medical Center, the local ethics committee of Necker-Enfants Malades Hospital, Paris, France and the Kyushu University Institutional Review Board for Human Genome/Gene Research. The patient from family A (referred to as patient 2 in ref. 23), patients from families B and C (referred to as pC and pS, respectively, in ref. 12), patient 2.2 from family D and the patients in family E (referred to as P4 and P8/P9 in ref. 5), as well as the three remaining ICFX cases (referred to as pN and P1 in ref. 12 and P4 in ref. 24) all show typical clinical features of ICF syndrome including hypo- or agammaglobulinemia in the presence of B cells, combined with the classical cytogenetic abnormalities involving chromosomes 1, 9 and 16, and hypomethylation of α-satellite DNA in addition to Sat II hypomethylation. The critical features of all patients are summarized in Supplementary Table 1; further details can be found in previous descriptions of these patients^2^,^5^,^6^,^12^,^23^,^24^.

### Gene identification by homozygosity mapping and sequencing

Homozygosity mapping was performed using the Sentrix HumanHap-300 Genotyping BeadChips (Illumina). To this end, 750-ng genomic DNA was processed with the Infinium II Whole-Genome Genotyping Assay (Illumina). After DNA amplification, fragmentation, precipitation and resuspension, DNA was applied to the BeadChip and incubated overnight, followed by enzymatic base extension, fluorescent staining of the beads and detection of fluorescent intensities using the BeadArray Reader (Illumina). To identify regions of homozygosity, B allele frequencies were assessed for all single-nucleotide polymorphisms using BeadStudio version 3.2 (Illumina). For a subset of patients, whole-exome sequencing was performed in the Neuromics project by deCODE Genetics (Reykjavik—Iceland) and analysed using deCODE Clinical Sequence Miner. Recessive analysis for multiple cases and controls and gene variant effect count (with variant effect predictor consequences moderate to high) were used to identify possible recessive mutations. For an additional set of patients, DNA libraries for whole-exome sequencing were constructed using the SureSelect Human All Exon V5 kit (Agilent Technologies) according to the manufacturer’s instructions. Sequencing was performed on the Illumina HiSeq2500 platform to generate 100 bp paired-end reads. Reads were mapped to the reference human genome (UCSC hg19) with the Burrows-Wheeler Alignment tool (BWA v0.7.4)^25^. Duplicate reads were removed by Picard (v1.87). We called SNVs and indels using the Genome Analysis Toolkit (GATK v2.5-2)^26^. Annotations of variants were made using Annotate Variation (ANNOVAR)^27^. For confirmation, relevant exons and flanking sequences of *CDCA7* and *HELLS* were amplified using standard PCR; products were purified and analysed using Sanger sequencing. Sequence tracks were analysed and visualized using ContigExpress (Vector NTI, Invitrogen-Life Technologies). PCR primers are listed in Supplementary Table 2.

### Cycloheximide treatment and RT–PCR analysis

Early-passage (*P*<6) patient-derived primary fibroblasts were maintained in DMEM F12 (31331) supplemented with 20% heat-inactivated fetal calf serum, 1% penicillin–streptomycin, 1% sodium pyruvate and 1% HEPES (all Invitrogen-Life Technologies). Cells were treated with 250 μg ml^−1^ cycloheximide (dissolved in ethanol, 01810, Sigma-Aldrich) for 4 h using equal volumes of ethanol as control. After treatment, cells were harvested in Qiazol lysis reagent and RNA was isolated using the miRNeasy mini kit (both Qiagen) all according to the manufacturer’s instructions. Total RNA (2 μg) was used for random primed cDNA synthesis using the RevertAid first-strand cDNA synthesis kit (Thermo Scientific). Transcripts were amplified using standard PCR with primers listed in Supplementary Table 2, separated by standard gel electrophoresis and sequenced by Sanger’s sequencing.

### Knockdown of gene expression in MEFs

The use of animal work has been reviewed by the Animal Experimentation Ethical Committee Buffon (CEEA-40), Paris, France, and was approved under the number CEB-06-2012. Female C57BL/6N pregnant mice, aged 3–6 months, were killed at 12.5 days post conception, and individual MEF clones were isolated from each embryo of the litter. Primary MEFs were cultivated for no more than two to three passages in complete media (DMEM glutamax supplemented with 10% fetal bovine serum, 100 U ml^−1^ penicillin, 100 μg ml^−1^ streptomycin, all from Life Technologies). Two rounds of transfection with synthetic short interfering RNAs (siRNAs) at a final concentration of 20 nM were performed using Interferin transfection reagent (Polyplus transfection) following the manufacturer’s instructions: a first round on MEFs in suspension and the second when cells were allowed to adhere to the plate. Cells were collected after 48 h for genomic DNA extraction. Sequences of siRNAs used in the study are listed in Supplementary Table 2.

### Satellite DNA methylation analysis using Southern blot analysis

Satellite II and α-satellite methylation in whole-blood DNA was analysed using Southern blot analysis of 2 μg genomic DNA digested with methylation-sensitive restriction enzymes HhaI for analysis of Sat-α or BstBI for analysis of Satellite II repeats. Both enzymes were purchased from Fermentas. After overnight digestion, DNA was separated by electrophoresis using 0.8% agarose gels. DNA was transferred overnight to Hybond-N+ membranes (GE Healthcare) and hybridized with a radioactive probe recognizing the α-satellite and satellite II repeats, respectively. Signals were detected by phosphoimaging or by exposure to X-ray films. For analysis of murine satellite repeats, genomic DNA was extracted and purified from MEFs using the NucleoSpin Tissue kit (Macherey-Nagel) according to the manufacturer’s instructions. The DNA pellet was eluted in Tris EDTA buffer containing 20 μg ml^−1^ RNAse A. Genomic DNA from MEFs (500 ng) was digested with 20 units of MspI or HpaII (New England Biolabs) for 16 h to analyse the DNA methylation patterns of centromeric minor satellite repeats. The digested DNA fragments were separated by electrophoresis using 1% agarose gels and transferred overnight to Hybond-N+ membranes (GE Healthcare) in 20 × SSC. After ultraviolet crosslink, the membranes were pre-hybridized in 6 × SSC, 5 × Denhardt and 0.1% SDS and then hybridized with 32P-labelled minor satellite oligonucleotide probe: (5′-ACATTCGTTGGAAACGGGATTTGTAGAACAGTGTATATCAATGAGTTACAATGAGAAACAT-3′).

Pre-hybridization and hybridization were carried out at 42 °C for 1 h. The membranes were washed three times in 6 × SSC and 0.1% SDS at 37 °C and signals were detected by phosphorimaging using FLA 7000 phosphorimager (Fuji). Uncropped scans of the Southern blots are presented in Supplementary Figs 4 and 5.

### Quantification of knockdown efficiency using qRT–PCR

Total RNA from MEFs was isolated using TRIzol Reagent (Life Technologies) according to the manufacturer’s instructions. Contaminant genomic DNA was eliminated with TURBO DNA-free kit (Ambion). Reverse transcription was carried out using 1 μg DNA-free RNA and 50 μM random hexamers, 20 U of RNase Out and 100 U of RevertAid reverse transcriptase (Life Technologies). Complementary DNA reactions were used as templates for PCR reactions. Real-time PCR was performed using the light cycler-DNA MasterPLUS SYBR Green I mix (Thermo Scientific) supplemented with 0.5 μM of specific primer pairs (listed in Supplementary Table 2). Real-time qPCRs were run on a light cycler rapid thermal system (Light Cycler 480 2.0 Real time PCR system, Roche) with 20 s of denaturation at 95 °C, 20 s of annealing at 60 °C and 20 s of extension at 72 °C for all primers, and analysed by the comparative CT (▵*C*_t_) method.

## Additional information

**How to cite this article:** Thijssen, P. E. *et al.* Mutations in *CDCA7* and *HELLS* cause immunodeficiency–centromeric instability–facial anomalies syndrome. *Nat. Commun.* 6:7870 doi: 10.1038/ncomms8870 (2015).

## Supplementary Material

Supplementary Information Supplementary Figures 1-5 and Supplementary Tables 1-2
