# Corrigendum: Mutations in *CDCA7* and *HELLS* cause immunodeficiency–centromeric instability–facial anomalies syndrome

**DOI:** 10.1038/ncomms12003

**Published:** 2016-06-22

**Authors:** Peter E. Thijssen, Yuya Ito, Giacomo Grillo, Jun Wang, Guillaume Velasco, Hirohisa Nitta, Motoko Unoki, Minako Yoshihara, Mikita Suyama, Yu Sun, Richard J. L. F. Lemmers, Jessica C. de Greef, Andrew Gennery, Paolo Picco, Barbara Kloeckener-Gruissem, Tayfun Güngör, Ismail Reisli, Capucine Picard, Kamila Kebaili, Bertrand Roquelaure, Tsuyako Iwai, Ikuko Kondo, Takeo Kubota, Monique M. van Ostaijen-Ten Dam, Maarten J. D. van Tol, Corry Weemaes, Claire Francastel, Silvère M. van der Maarel, Hiroyuki Sasaki

Nature Communications 6: Article number: 7870 10.1038/ncomms8870 (2015); Published: 07282015; Updated: 06222016

In Fig. 1b of this Article, the sex of patient 2.1 in family A is incorrect, and should be depicted as male. The correct version of this figure appears below.


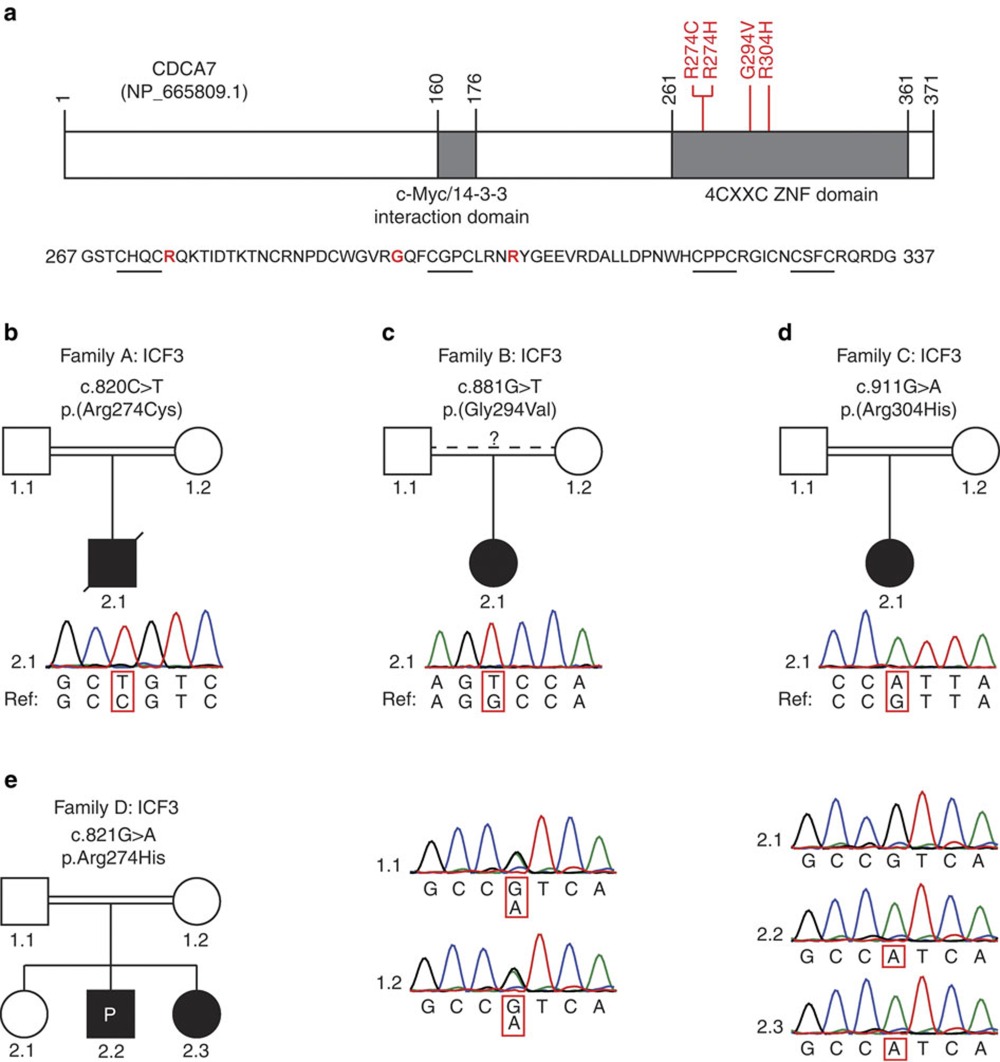


**Figure 1**

