# Supplementary material for: Mutations in CDCA7 and HELLS cause immunodeficiency–centromeric instability–facial anomalies syndrome
Source: Nat Commun. 2015 Jul 28;6:7870. doi: 10.1038/ncomms8870 (PMC4519989; doi:10.1038/ncomms8870)
Supplement: Supplementary Information — Supplementary Figures 1-5 and Supplementary Tables 1-2 [file ncomms8870-s1.pdf]

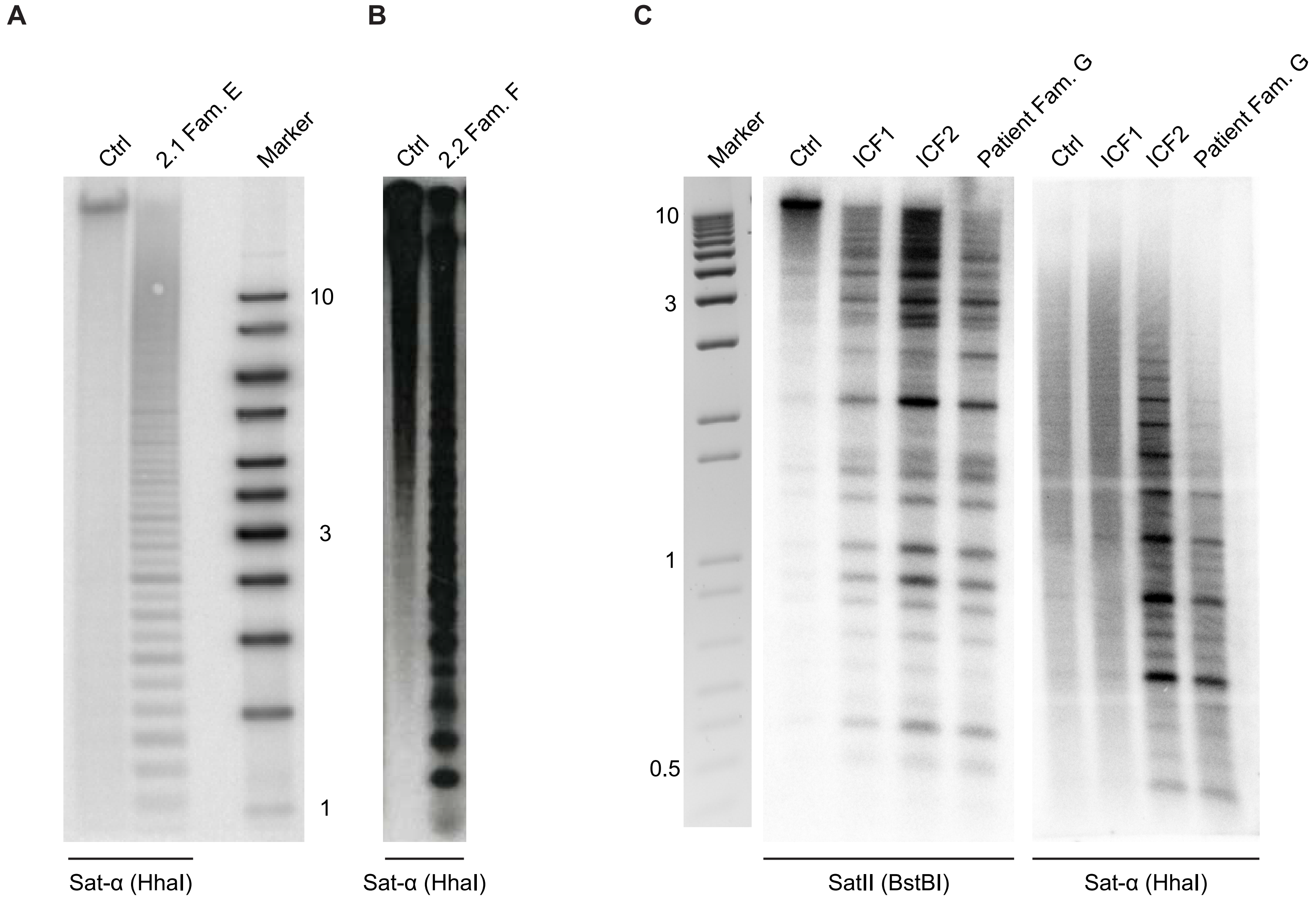

Supplementary Figure 1: Repeat hypomethylation in ICF patients from families E, F and G
(a, b) Sat-α methylation analysis using HhaI digestion followed by Southern blot analysis of healthy controls and patients 2.1 from family E and 2.2 from family F showed hypomethylation in the patient derived samples. **(c)** SatII (BstBI) and Sat-α (HhaI) methylation analysis by Southern blot analysis in DNA derived from a healthy control, an ICF1 patient, an ICF2 patient and the patient from family G confirmed SatII hypomethylation in all ICF samples and Sat-α hypomethylation in ICF2 and ICFX samples. Molecular weights of the DNA size markers are in kilo-basepairs.

```
CDCA7   261  YNRSLGSTCHQCRQKTIDTKTNCRNPDCWGVRGQFCGPCLRNRYGEEVRDALLDPNWHCPPCRGICNCSFCRQRDGRCATGVLVYLAKYHGFGNVHAYLK  361
             Y++ LG+TCHQCRQKTIDTKT CRN  C GVRGQFCGPCLRNRYGE+VR ALLDP+W CPPCRGICNCS+CR+RDGRCATG+L++LAK++G+ NV  YL+
CDCA7L  345  YDKVLGNTCHQCRQKTIDTKTVCRNQGCCGVRGQFCGPCLRNRYGEDVRSALLDPDWVCPPCRGICNCSYCRKRDGRCATGILIHLAKFYGYDNVKEYLE  444
```

Supplementary figure 2: Aligment of the 4-CXXC domains of CDCA7 and CDCA7L
Alignment using blastp (NCBI) showed 76% sequence identity between the 4-CXXC domain of CDCA7 with that of CDCA7L. Identified mutations are highlighted in red.

a

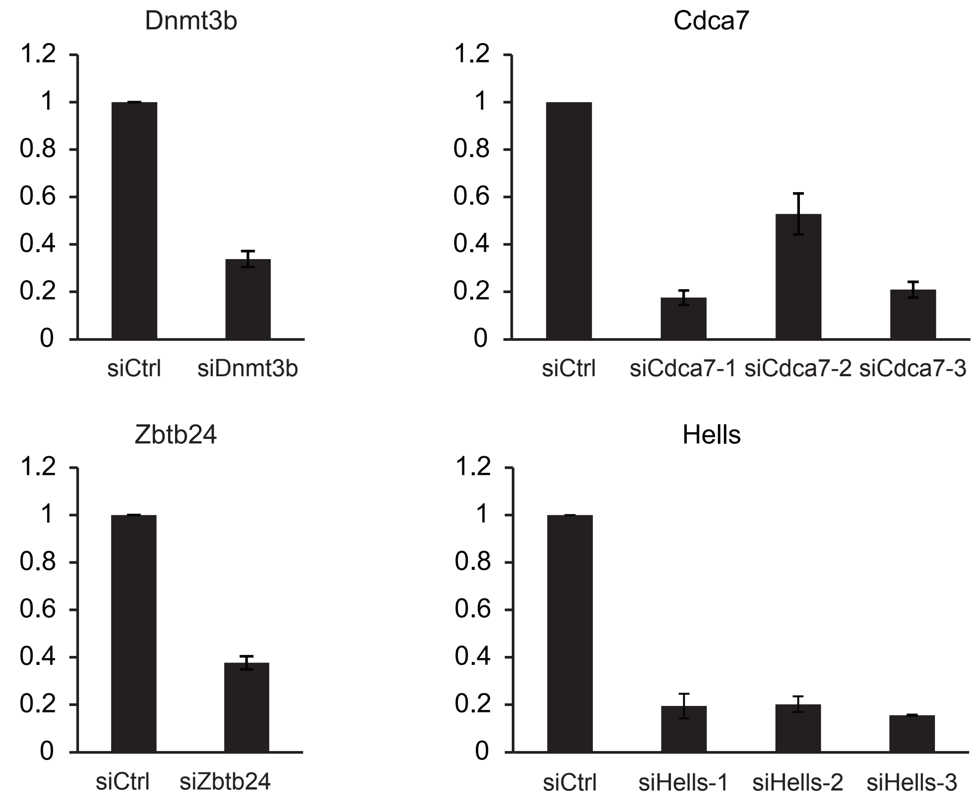

b

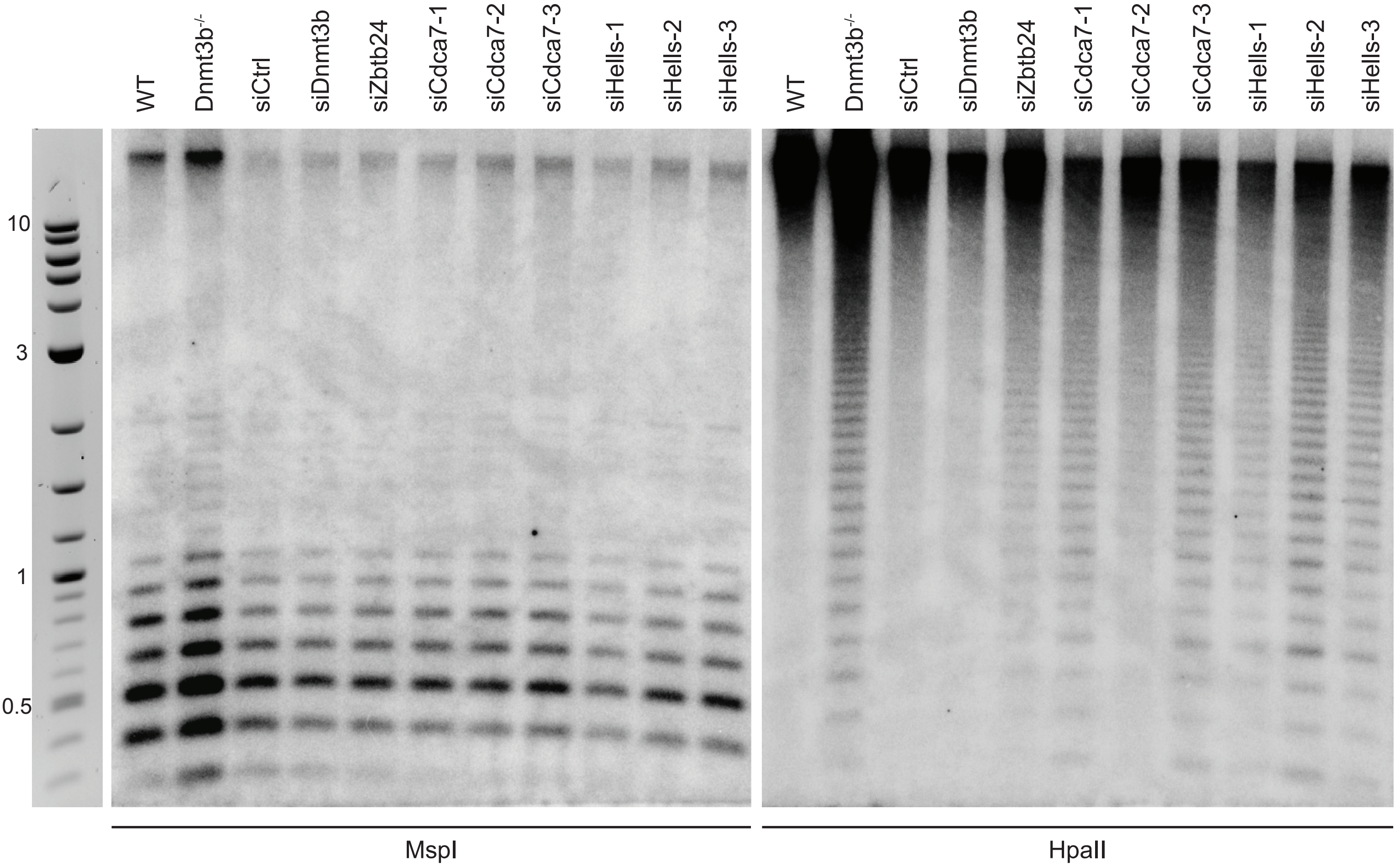

Supplementary figure 3: confirmation of minor satellite hypomethylation through siRNA mediated knockdown in wildtype MEFs (a) qRT-PCR quantification of mRNA expression of the indicated transcript upon knockdown using the indicated siRNAs, siCtrl levels are set to 1, error bars indicate SEM of average knockdown levels of at least two independent experiments. **(b)** Replication and extension of the data in figure 2g showing minor satellite DNA hypomethylation in wild type MEFs upon knockout of Dnmt3b and knockdown of Zbtb24, Cdca7 and Hells with different siRNA's. Molecular weights of the 2-Log DNA size marker are in kilo-basepairs.

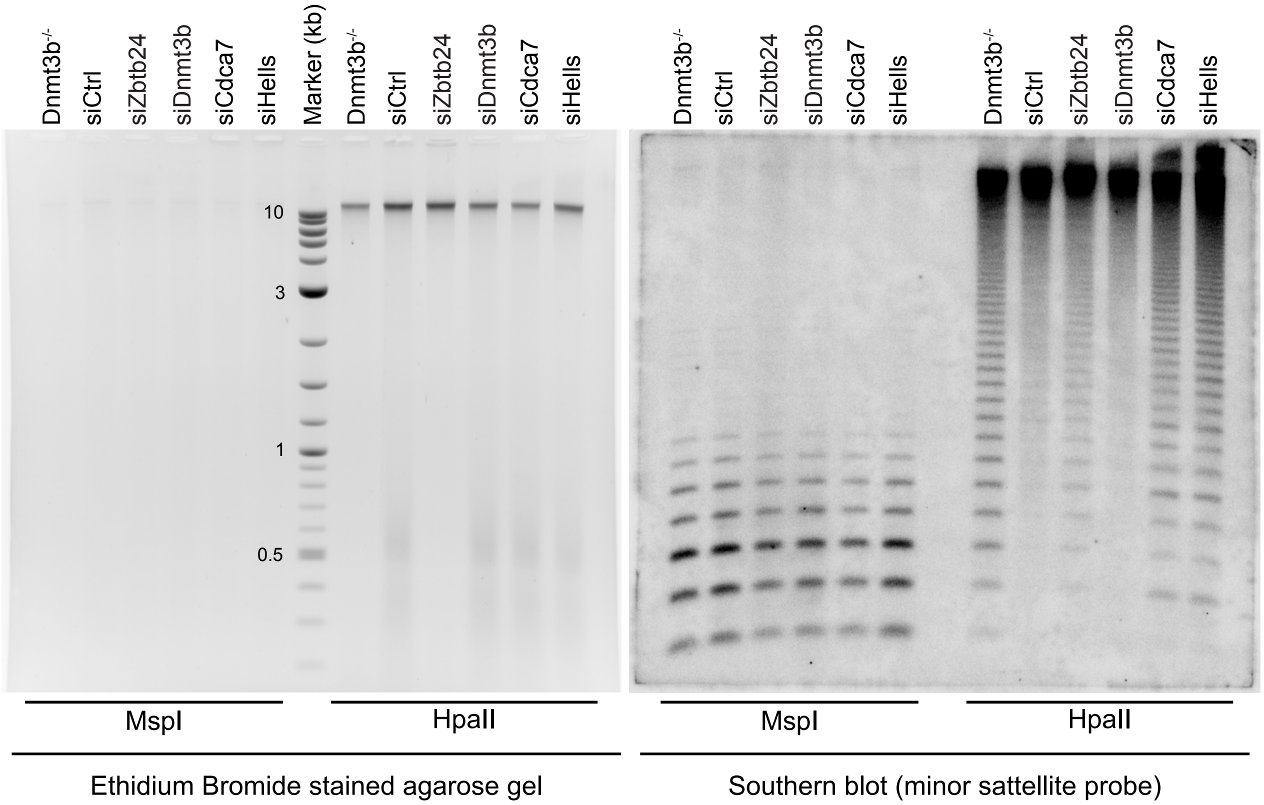

Supplementary Figure 4: Uncropped images of agarose gels and Southern blots
Uncropped images corresponding to Figure 2g. Left panel: ethidium bromide stained agarose gel, right panel: Southern blot probed for minor satellites. Molecular weights of the 2-Log DNA size marker are in kilo-basepairs.

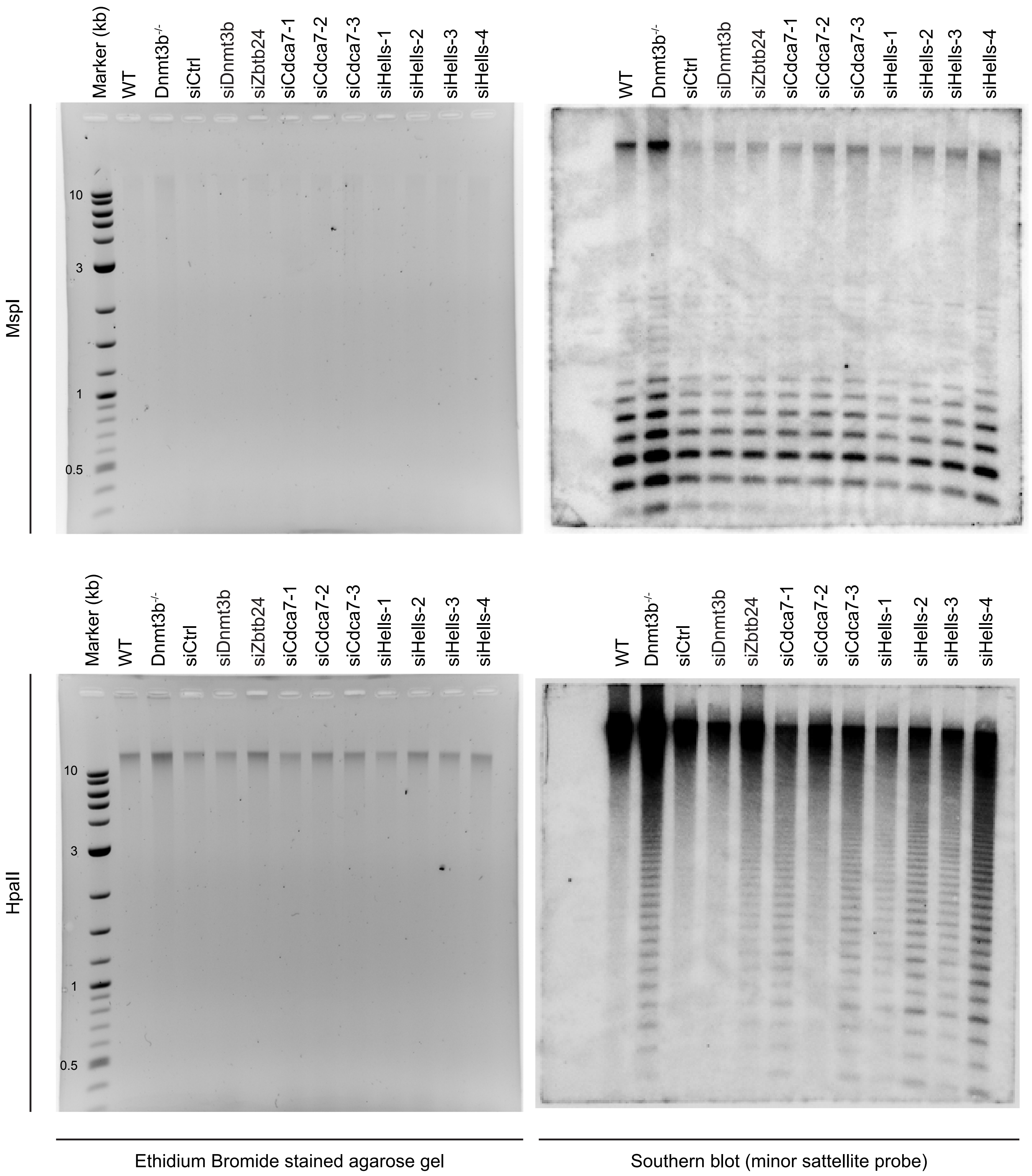

Supplementary Figure 5: Uncropped images of agarose gels and Southern blots
Uncropped images corresponding to Supplementary Figure 3b. Left panels: ethidium bromide stained agarose gels, right panels: Southern blots probed for minor satellites. Upper panels: MspI digestion, lower panels: HpaII digestion. Molecular weights of the 2-Log DNA size marker are in kilo-basepairs.

Supplementary Table 1: demographic and clinical characteristics of patients

ICF type	ICF3	ICF3	ICF3	ICF3	ICF3	ICF4	ICF4	ICF4	ICF4	ICF4	ICF1	ICF2
Family (individual)	A	B	C	D (2.2)	D (2.3)	E (2.1)	E (2.2)	F	G	H	*n*= 23	*n*= 13
Gene defect	*CDCA7*	*CDCA7*	*CDCA7*	*CDCA7*	*CDCA7*	*HELLS*	*HELLS*	*HELLS*	*HELLS*	*HELLS*	*DNMT3B*	*ZBTB24*
Mutation Paternal allele	c.820C>T	c.881G>T	c.911G>A	c.821G>A	c.821G>A	c.2096A>G	c.2096A>G	c.2283_2286delGTCT	c.2400_2402delGTT	c.610A>T		
	p.(Arg274Cys)	p.(Gly294Val)	p.(Arg304His)	p.Arg274His	p.Arg274His	p.Gln699Arg	p.Gln699Arg	p.(Ser762Argfs*4)	p.(Leu801del)	p.(Lys204*)		
ExAC allele frequency	0	0	8.24E-06	8.24E-06	8.24E-06	0	0	0	8.26E-06	0		
Provean score	-7.306	-8.219	-4.566	-4.566	-4.566	-3.825	-3.825	N/A	-9.303	N/A		
PolyPhen-2 score	prob. damag.	prob. damag.	prob. damag.	prob. damag.	prob. damag.	prob. damag.	prob. damag.	N/A	N/A	N/A		
Mutation Maternal allele	c.820C>T	c.881G>T	c.911G>A	c.821G>A	c.821G>A	c.370+2T>A	c.370+2T>A	c.2283_2286delGTCT	c.2400_2402delGTT	c.374_381dup		
	p.(Arg274Cys)	p.(Gly294Val)	p.(Arg304His)	p.Arg274His	p.Arg274His	skip ex5	skip ex5	p.(Ser762Argfs*4)	p.(Leu801del)	(p.Lys128*)		
ExAC allele frequency	0	0	8.24E-06	8.24E-06	8.24E-06	0	0	0	8.26E-06	0		
Provean score #	-7.306	-8.219	-4.566	-4.566	-4.566	N/A	N/A	N/A	-9.303	N/A		
PolyPhen-2 score *	prob. damag.	prob. damag.	prob. damag.	prob. damag.	prob. damag.	N/A	N/A	N/A	N/A	N/A		
Sex	M	F	F	M	F	F	M	F	M	F		
Birth year	1982	1998	1978	2005	2012	2000	2007	2002	1999	2010		
Deceased (age in yrs)	yes (26)							yes (1.2)				
Consanguineous (reported)	yes	suspected	yes	yes	yes	no	no	unknown	yes	unknown		
Origin	Turkish	French	French	Turkish	Turkish	English	English	Japanese	French	Italian		
Birth weight (grams)	1960	730	1820	2800	2250	2400	3100	1770	3830	900	<p3 (3/20) ‡	<p3 (1/7) ‡
Motor developmental delay	yes	yes		no	no	no	yes			yes	9/16	7/8
Mental retardation	yes	yes	no	no	no	yes (mild)	no		yes	yes	9/20	13/13
Facial anomalies	yes	yes	no	yes	yes	yes	yes	yes	yes	yes	21/22	13/13
Gastrointestinal problems	yes	yes	yes	no	yes	yes	no	no	no	no	7/14	2/6
Infections	otitis<br>pneumonia	otitis<br>pneumonia<br>myocarditis<br>peritonitis	otitis<br>pneumonia<br>mastoiditis<br>meningitis	otitis	pneumonia<br>Candida	pneumonia<br>Pneumocystis	no	pneumonia<br>Pneumocystis	otitis<br>pneumonia	bronchitis	8/13<br>16/16<br>Pneumocystis (n=2)<br>Candida (n=4)	2/6<br>5/7<br>Pneumocystis (n=2)<br>Candida (n=2)
Cytogenetic abnormalities<br>Chr 1, 9 and 16	Stretching<br>Multiradial<br>configuration	Stretching<br>Associations<br>Rosettes<br>Branching<br>Micronuclei<br>Decondensation	Stretching	Stretching<br>Breaking<br>Decondensation		Isochromosomes<br>Multiradial<br>configuration		Stretching<br>Associations<br>Rosettes<br>Branching<br>Micronuclei<br>Decondensation	Translocations<br>Deletions	Rosettes		
α-satellite hypomethylation	N/A	Yes (ref. 13)	Yes (ref. 13)	Yes (ref. 7)	N/A	Yes (this study)	N/A	Yes (this study)	Yes (this study)	N/A	No	Yes
Treatment						stem cell<br>transplantation	stem cell<br>transplantation					
IgG (g/l)	2.8	0.31	2	2.32	1.11	< 0.33	8.05 (maternal)	0.23	0.91	IVIG		
IgA (g/l)	< 0.04	0.05	0.27	< 0.07	0.06	< 0.07	< 0.3	0.06	< 0.04	< 0.04		
IgM (g/l)	0.23	< 0.06	0.25	0.03	0.04	0.10	< 0.22	0.05	0.04	< 0.04		

#: Protein Variation Effect Analyzer (provean.jcvi.org/)
*: Polymorphism Phenotyping v2 (genetics.bwh.harvard.edu/pph2/)
‡: Birthweight less than the third percentile for gestational age

Supplementary table 2: List of primers and siRNAs

List of primers:

human exons:	**Fw primer:**	**Rev primer:**	**Mutation:**
CDCA7 exon 8	GGACATTCTGTAAATATGCCATTTC	TCCACCCCTTCACCAATG	c.820C>T; c.821G>A
CDCA7 exon 8	GCAAGCCATTGTGATTGGTT	CATGTGGCTGAATGAATTGC	c.881G>T; c.911G>A
HELLS exon 5-6	GGCTCAATTGCAGGAAACAT	TTAAAACCTACATTTTACTCAACATCA	c.370+2T>A<br>c.374_381dup
HELLS exon 8	GCTTCCCAAAGTGCTGTGAT	GCCATAAAAGTGCTGGAAGC	c.610A>T
HELLS exon 20	CCTGGCTAGATCATTCATGTTG	ACTCGGCCCAGGCTGTAATA	c.2096A>G
HELLS exon 21-22	CCGTCCTTCTTAATGCAAGC	CATGTGGCTGAATGAATTGC	c.2283_2286delGTCT<br>c.2400_2402delGTT

Human transcripts:	**Fw primer:**	**Rev primer:**
HELLS ex3-5	TGACGAAAATGGAACAGCAA	CTCTTTTGACATGACCTCTGAAAT

Murine transcripts:	**Fw primer:**	**Rev primer:**
Dnmt3b	CGCCACCATGTGCAGGAGTAC	GACGCTCTTAGGTGTCACTTCTTCC
Zbtb24	GCTGGAGCACATGAGCCTG	TTGGCACTGGAGTCAGAGAAG
Cdca7	GACAGCTACATGAACGACGATG	GATGACATGTAGAACCCAGAGAG
Hells	ACACGAGCTGGTGGTTTAGG	GCCTGGAGATCAGACTGAGG

List of siRNA's

Target gene:	**siRNA sequence (5' -> 3')**
Dnmt3b	GGCUGUUCAGCCAGCACUUUAAU
Zbtb24	GAAGCCAUUUACUUGUGAA
Cdca7-1	AUGACAGUUGUGACAGCUUU
Cdca7-2	CCUCUUACCAGGUCAAGGU
Cdca7-3	AGGAGGAAGAGGAUAAGUA
Hells-1	GAGAAGUUGAUAAUCCAUA
Hells-2	GUCUACACUUCCUAACUGG
Hells-3	GUCUGAUGUUGCUCUUGAA
